# Stress Degradation Studies of Tebipenem and a Validated Stability-Indicating LC Method

**DOI:** 10.1007/s10337-012-2331-4

**Published:** 2012-10-05

**Authors:** Judyta Cielecka-Piontek, Przemysław Zalewski, Bolesław Barszcz, Kornelia Lewandowska, Magdalena Paczkowska

**Affiliations:** 1 Department of Pharmaceutical Chemistry, Faculty of Pharmacy, Poznan University of Medical Sciences, Grunwaldzka 6, 60-780 Poznań, Poland; 2 Department of Molecular Crystals, Institute of Molecular Physics Polish Academy Sciences, Smoluchowskiego 17, 60-179 Poznań, Poland

**Keywords:** Column liquid chromatography, Stress degradation studies, HOMO–LUMO, Intramolecular charge transfer, Tebipenem

## Abstract

A inexpensive and rapid isocratic LC method has been developed for the quantitative determination of tebipenem—a new β-lactam antibiotic. Stress degradation studies were performed on tebipenem in acidic (0.2 N hydrochloric acid) and basic (0.02 N sodium hydroxide) solutions, in a solution with oxidizing agent (3 % hydrogen peroxide), and in the solid state, during thermolysis and photolysis. For a chromatographic separation of tebipenem and its degradation products, a C-18 stationary phase and 12 mM ammonium acetate-acetonitrile (96:4 v/v) were used. A quantitative determination of tebipenem was carried out by using a PDA detector at 298 nm, with a flow rate of 1.2 mL min^−1^. The linear regression analysis for the calibration plots showed a good linear relationship (*r* = 0.999) in the concentration range 0.041–0.240 mg mL^−1^. The method demonstrated good precision (1.14–1.96 % RSD) and recovery (99.60–101.90 %). The limits of detection and quantitation were 9.69 and 29.36 μg mL^−1^, respectively. The analysis of tebipenem reactivity was supported by quantum chemical calculations based on the density functional theory (DFT). The analysis of the electron density of the HOMO and LUMO of tebipenem suggested the possibility of electron transport in the molecule during the degradation of bi-cyclic 4:5 fused penem rings.

## Introduction

Tebipenem is the active form of tebipenem pivoxil, a novel oral carbapenem antibiotic that has been approved for the treatment of bacterial diseases caused by G-positive and G-negative bacteria in pediatric patients [1]. Tebipenem similarly to other CH_3_ carbapenems contains the bi-cyclic 4:5 fused β-lactam and pyrrolidine rings, a *trans*-1-hydroxyethyl substituent at C-6 and a methyl group at C-4. The presence of a 1-[(1,3-thiazolin-2-yl)azetidin-3-yl]thio substituent at C-2 is the differentiating feature of the molecule which influences the antibacterial activity of tebipenem and can affect its chemical stability [2]. In a molecule of tebipenem, similarly to other carbapenem analogs, the significant instability of the β-lactam ring is a consequence of the presence of a bi-cyclic 4:5 fused ring, represented by the fusion of the β-lactam ring and the pyrrolidine moiety [3]. During the degradation of carbapenems depending on stress factors (solvents, pH, drug concentration, temperature, time) and their chemical structures, various degradation products are formed [4].

Research reports provide only data on antibacterial efficiency, crystallographic structures of tebipenem complexed with penicillin-binding proteins, and pharmacokinetic parameters [5–8]. To the best of our knowledge, no LC method for the determination of tebipenem in the presence of its degradation products in the pharmaceutical matrix has so far been reported. The International Conference on Harmonization (ICH) guidelines require the development of stability-indicating methods (SIAMs) for drug assays in stability tests. SIAMs should be suitable for drug determination during hydrolysis (at various pH), oxidation, photolysis, and thermal degradation [9].

During stability studies, the prediction of the pathways of degradation and places in molecules which are more susceptible to degradation can by supported by quantum-chemical calculations [10]. That is especially important for stability studies of novel compounds when the main analytical tools are chromatographic procedures. Moreover, the analysis of electron density of the HOMO and the LUMO shows location of electrons, consequently offers an indication of molecule reactivity.

The aim of the work was twofold: to develop a stability-indicating LC method for determination of tebipenem to be used for stability assessment and for quality control during the manufacturing process, and to establish the interdependence between the reactivity of tebipenem and stress factors.

## Experimental

### Chemicals, Reagents, and Solutions

The tebipenem substance (purity >98 %) were supplied by Pharmachem International (China). Tebipenem is a white to slightly yellowish sterile crystalline powder. All other chemicals and solvents were obtained from Merck (Germany) and were of analytical grade. High quality pure water was prepared by using the Millipore purification system (model Exil SA 67120; Millipore, Molsheim, France).

### HPLC Instrumentation and Chromatographic Conditions

The chromatographic separation and quantitative determination were performed using a high performance liquid chromatograph containing a Shimadzu pump, model LC-6A, a UV–VIS detector SPD-6AV (Shimadzu), and a Rheodyne 7120 with a 50-μl loop. As the stationary phase, a Lichrospher RP-18 column, 5-μm particle size, 250 × 4 mm (Merck, Darmstadt, Germany) was used. The mobile phase consisted of 4 volumes of acetonitrile and 96 volumes of ammonium acetate, 12 mmol L^−1^. The flow rate of the mobile phase was 1.2 mL min^−1^. The wavelength of the UV–VIS detector was set at 298 nm. Photodegradation stability studies were performed using Suntest CPS^+^ (Atlas^®^) with filter Solar ID65. For analysis of homogeneity peak of forced degradation samples, the photodiode array detector (L-7455; Merck) was used in scan mode with a scan range of 200–600 nm.

### Theoretical Studies

In order to interpret the initial geometry of molecule and spatial electron distribution of molecular orbitals: HOMO (the highest occupied molecular orbital) and LUMO (the lower unoccupied molecular orbital), quantum chemical calculations were performed. All the calculations were made by using the Gaussian 03 package [11]. Quantum chemical calculations were optimized by means of a density functional theory (DFT) method with the B3LYP hybrid functional and 6-31G(d,p) basis set.

### Method Validation

HPLC method was validated according to International Conference on Harmonization Guidelines [9].

### Selectivity

The selectivity was examined for non-degraded and degraded samples: the solutions of tebipenem after stress conditions of hydrolysis (0.2 N HCl, *T* = 303 K; 0.02 N NaOH, *T* = 298 K), photolysis (sunlight), oxidation (3 % H_2_O_2_, *T* = 298 K), and thermal degradation at increased relative humidity (RH = 76.5 %, 343 K) and at dry air (RH = 0 %, 373 K).

### Linearity

The calibration plots *P* = *f*(*c*) were obtained in the concentration range 0.041–0.240 μg mL^−1^, *P* is the peak area of tebipenem.

### Accuracy, as Recovery Test

The accuracy of the method was determined by recovering tebipenem from the placebo. The recovery test was performed at three levels: 80, 100, and 120 % of the nominal concentration of tebipenem during degradation studies. Three samples were prepared for each recovery level. The solutions were analyzed and the percentage of recoveries was calculated from the calibration curves.

### Precision

Precision of the assay was determined in relation to repeatability (intra-day) and intermediate precision (inter-day). In order to evaluate the repeatability of the methods, six samples were determined during the same day for three concentrations of tebipenem. Intermediate precision was studied comparing the assays performed on two different days.

### Limits of Detection and Quantification

The limits of detection (LOD) and quantification (LOQ) parameters were determined from the regression equation of tebipenem: LOD = 3.3 *S*_*y*_/*a* and LOQ = 10 *S*_*y*_/*a*, where *S*_*y*_ is a standard error and *a* is the slope of the corresponding calibration curve.

### Robustness

The robustness of the procedure was evaluated after changing the following parameters: the composition of the mobile phase (content of acetonitrile in the range 4–6 %), the wavelength of absorption (in the range 295–305 nm), and temperature (25 ± 2 °C). For each parameter change, its influence on the retention time, peak, and area (height and width) was evaluated.

## Procedure for Stability Studies of Tebipenem

### Acid and Base Hydrolysis

The degradation of tebipenem in aqueous solutions was studied at 303 K in hydrochloric acid (0.2 N) and in sodium hydroxide (0.05 N) at room temperature. The ionic strength of all solutions was adjusted to 0.5 mol L^−1^ with a solution of sodium chloride (4.0 mol L^−1^). Degradation was initiated by dissolving an accurately weighed 5.0 mg of tebipenem in 25.0 ml of the solution equilibrated to desired temperature in stoppered flasks. At specified times, samples of the reaction solutions (1.0 mL) were instantly cooled with a mixture of ice and water, and neutralized.

### Oxidative Degradation

An amount of 5.0 mg of tebipenem was accurately weighed and dissolved in 5.0 mL of diluent (water), then 25.0 mL of 3 % H_2_O_2_ solution was added. Samples of reaction solutions were studied immediately.

### Thermal Degradation

Samples of 5.0 mg of tebipenem were weighed into 5-ml vials. In order to achieve the degradation of tebipenem in solid state, the samples were kept in heat chambers at 373 K, at RH = 0 %, and at 343 K, at RH = 76.5 %. At specified time intervals, determined by the rate of degradation, the vials were removed, cooled to room temperature, and their contents were dissolved in distilled water. The obtained solutions were quantitatively transferred into measuring flasks and diluted with water to 25.0 mL.

### UV Degradations

Samples of 5.0 mg of tebipenem were accurately weighed and then exposed to sunlight (10,000 lux) for a period of 48 h. The samples were dissolved in distilled water to 25.0 mL.

## Results and Discussion

### Method Development

**Fig. 1:**
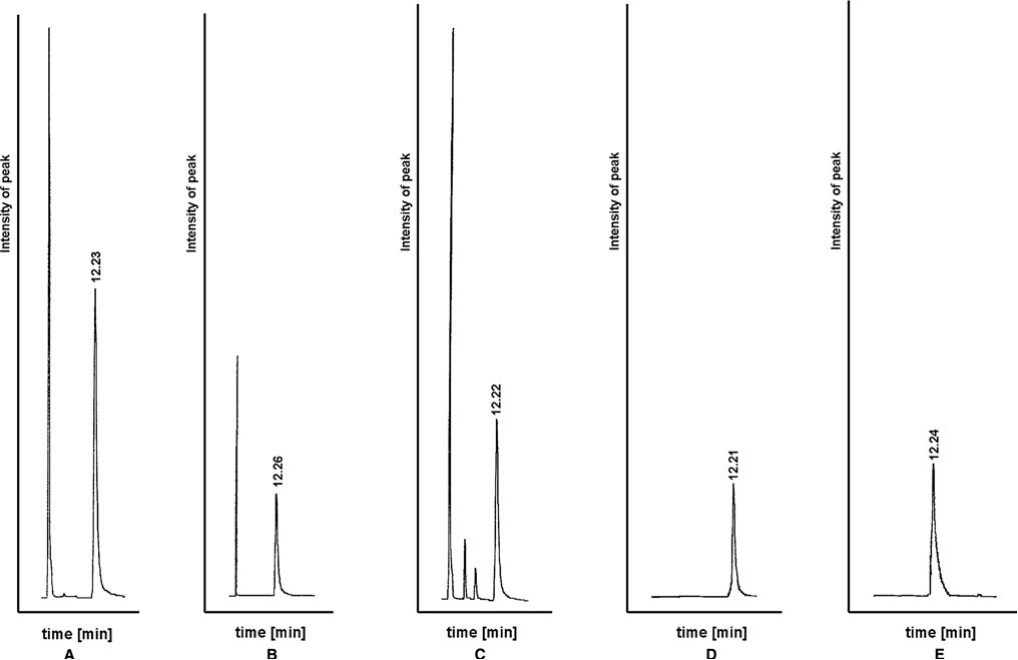
Chromatograms: solution of tebipenem (**a**), solution of tebipenem in HCl (0.2 N) after incubation for 3 min at 303 K (**b**), solution of tebipenem in H_2_O_2_ (3 %) after incubation for 2 min at 298 K (**c**), solution of tebipenem after its incubation in the solid state at increased relative humidity (RH = 76.5 %, 45 min) (**d**), and solution of tebipenem after its incubation in the solid state in dry air (RH = 0 %, 72 h) (**e**), *t*_R_ ~ 12.32 min, tebipenem, other *t*_R_-related products

The main target of chromatographic determination of tebipenem was to obtain the separation of close eluting peaks originating from related substance and degradation products. The pathways of degradation of carbapenem analogs and kinds of forming degradation products depend on the affecting factors. The literature reports that, depending on affecting factors during the CH_3_-carbapenems analogs degradation, the structures with an opened β-lactam ring or dimmers were formed [4], respectively, in aqueous solutions during acid–basic hydrolysis and oxidization, as well as in solid state, during thermolysis and photolysis. For that reason, the establishment of selectivity of determination is the crucial validation parameter. Satisfactory chromatographic separation between tebipenem and degradation products was achieved by using an LiChrospher column (RP-18, 5 μm particle size, 250 × 4 mm) and ammonium acetate (12 mmol L^−1^):acetonitrile (96:4 v/v) as mobile phase. The column temperature was maintained at 25 °C and the wavelength of detection was 298 nm. The injection volume was 50 μL. The purity of the tebipenem peak in degraded samples was checked by assessment of spectrophotometric peak homogeneity. The typical chromatograms of tebipenem and degradation products are shown in Fig. 1. During optimization of the chromatographic method, the most significant factor was content of organic solvents. Other factors (length of column, temperature of analysis, concentration of ion-pair fraction) did not significantly influence the parameters of the method, while the content of the organic fraction was the important factor, determining the desired elution time of tebipenem. A mixture of ammonium acetate:acetonitrile (96:4 v/v) was selected as the optimum mobile phase due to the desired peak shape (peak area, asymmetry, tailing factor), baseline drift, time required for analysis, and cost of solvent. Under these conditions, the retention time and asymmetry factor were 12.32 ± 0.01 and 1.415 ± 0.02 min, respectively. As shown on the chromatograms, degradation products formed under the influence of oxidative factor were recorded, with an elution time shorter than for tebipenem. Stability-indicating analytical methods for the determination of meropenem, biapenem, and doripenem based on the similar kinds of compounds were chosen as mobile phase [12–15]. However, the introduction of a 1-[(1,3-thiazolin-2-yl)azetidin-3-yl]thio substituent at C-2 in the tebipenem molecule significantly lengthened its elution time compared to other CH_3_-carbapenems.

### Method Validation

The method was validated for parameters such as specificity, linearity, precision, accuracy, and robustness. The calibration curve was linear and described by the equation *y* = (197.69 ± 8.94) 10^4^ *x* (*n* = 10, *r* = 0.9993). The parameters of regression were calculated for *f* = *n *– 2 degrees of freedom and *α* = 0.05. The values *b*, calculated from the equation *y* = *ax* + *b,* were not significant. The percentage recovery of tebipenem was established at three levels, 80, 100, and 120 % of label claim of the substance and were 99.60 and 101.90 %, respectively. The %RSD values for intramediate precision were found to be 1.14–1.96 %, while the %RSD in determination of intermediate precision was 2.05 % (100 % of label claim). Under applied chromatographic conditions, the LOD and LOQ of tebipenem were 9.69 and 29.36 μg mL^−1^, respectively. A stock solution of tebipenem (0.20 mg mL^−1^) was prepared by dissolving an appropriate amount in diluents. Working solutions were stable when they were stored at room temperature and protected from light during 0.5 h. No significant changes in resolution, shapes, areas of peaks, and retention times were observed when the temperature of the column, concentration of the inorganic fraction of the mobile phase, and flow rate were modified. Modifications of the composition of the mobile phase: organic-to-inorganic component ratio resulted in the essential changes of retention time and resolution in determination of tebipenem. Validation parameters are demonstrated in Table 1. The system suitability parameters, including peak area (38,256 ± 10), retention time (12.32 ± 0.01 min), theoretical plates (2,720), and tailing factor (1.415 ± 0.02), were determined.

**Table 1:** Validation parameters of tebipenem

Label claim (%)/spiked concentration (μg mL^−1^)	Validation parameters	
	Intra-day precision (*n* = 6) (%)	Accuracy (*n* = 3) (%)
80/0.160	1.52	99.60
100/0.191	1.14	100.21
120/0.240	1.92	101.90

### Results of Stability Studies of Tebipenem

**Fig. 2:**
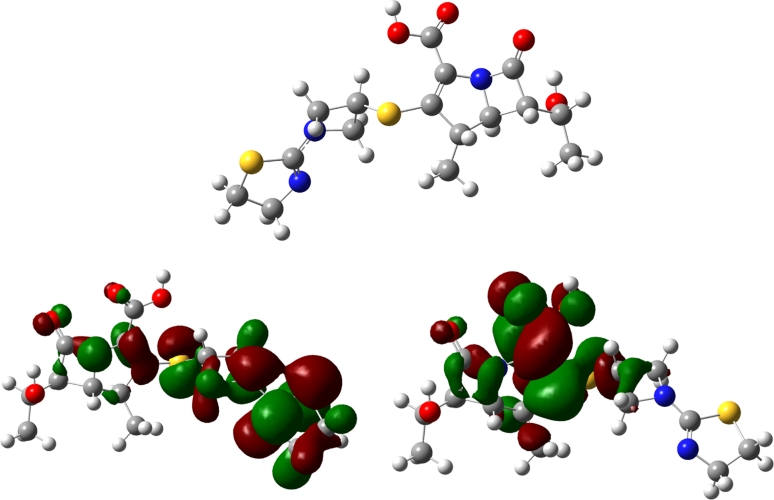
The initial geometry, LUMO and HOMO orbitals of tebipenem

The degradation of tebipenem was observed in solutions during stress studies in acidic and basic hydrolysis, under oxidizing conditions, and in the solid state at an increased relative humidity (RH = 76.5 %) and in dry air (RH = 0 %). No degradation was noted in the solid state during photolysis. Tebipenem was found to be the most sensitive to degradation in basic solutions (0.01 N NaOH, *T* = 298 K), to the extent that, upon contact with the basic factor, its total degradation occurred immediately. Under the influence of acidic (0.2 N HCl, *T* = 303 °C) and oxidating (3 % H_2_O_2_, *T* = 298 K) factors, degradation was delayed (approximately 40 % after 3 min.). The results of forced degradation in various media are summarized in Table 2. The chromatograms of solutions obtained after degradation under acidic, basic, and oxidizing conditions and in the solid state are shown in Fig. 1. It was found that, during the degradation of tebipenem, different degradation products were observed which has been demonstrated by previous studies. During degradation of other carbapenems, the kind of affecting factors also influenced the kind of degradation product [4]. A decrease in the content of tebipenem was recorded on the chromatograms of tebipenem after acidic and basic degradation. However, no peaks of degradation products occurred on the chromatograms. When tebipenem was degraded under the influence of oxidizing agents, the peaks of degradates appeared on the chromatograms. The lack of substituents containing the π-bond system chromophores in tebipenem appeared to be the limitation in the application of LC coupled with a PDA detector for the determination of tebipenem degradation products, and for predicting its degradation pathways. The current stability study of tebipenem was connected with the establishment of the initial geometry of its molecule and the spatial distribution of the molecular orbitals, HOMO and LUMO, which led to the suggestion of the extent of tebipenem reactivity. The presence of intra-ring stress connected with fusion 4:5 β-lactam and pyrrolidine rings, which was indicated during the analysis of the initial geometry of the tebipenem molecule, can be interpreted as a consequence of degradation under the influence of increased temperature (Fig. 2). The varying results of the influence of chemical affecting factors can be explained by analyzing the spatial distribution of the electron on the molecular orbitals of tebipenem, especially those of the HOMO and the LUMO (Fig. 2). The LUMO orbital localized on the fused 4:5 rings indicating the presence of atomic centers vulnerable to nucleophillic attacks. The lower susceptibility to degradation of tebipenem in dry air than at an increased relative humidity proved that the carbonyl carbon in the β-lactam ring is also targeted by nucleophilies in the solid state. Based on the significant delocalization of the HOMO orbital on the 1-[(1,3-thiazolin-2-yl)azetidin-3-yl]thio substituent, it is possible to assume that an electron transport in this part of the tebipenem molecule during acidic hydrolysis and oxidation is responsible for the reactivity of the molecule under such conditions. As a result, electron transport does not occur in the area of the fused 4:5 rings in the first stage of tebipenem degradation, allowing the detection of related products.

**Table 2:** Results of forced degradation studies

Stress conditions and time studies	Degradation (%) tebipenem	Stress conditions and time studies	Degradation (%) tebipenem
Acidic medium/0.2 N HCl/303 K/2 min	65.40	Thermolysis/RH = 76.5 %/343 K/45 min	50.45
Basic medium/0.05 N NaOH/RT/1 min	100.0	Thermolysis RH = 0 %/373 K/72 h	45.34
Oxidizing medium/3 % H_2_O_2_/RT/3 min	45.67	Photolysis/48 h	0

## Conclusions

An isocratic RP-LC method developed for quantitative analysis of tebipenem is precise, accurate, and specific, and may be useful for routine analysis in quality control and stability studies. In aqueous solutions, tebipenem is the most susceptible to degradation under the influence of alkalic factor. During degradation of tebipenem in the solid state, the humidity is the significant factor which caused the break of the β-lactam ring. Moreover, by combining the results of chromatographic studies with the findings of quantum chemical calculations, it was possible to propose an explanation of the changes in the reactivity of tebipenem depending on affecting factors.

## References

[1] Kato K, Shirasaka Y, Kuraoka E, Kikuchi A, Iguchi M, Suzuki H, Shibasaki S, Kurosawa T, Totsuka K (2010). Mol Pharm.

[2] Cielecka-Piontek J, Michalska K, Zalewski P, Jelińska A (2011). Cur Pharm Anal.

[3] Zhanel G, Wiebe R, Dilay L, Thomson K, Rubinstein E, Hoban D, Noreddin A, Karlowsky J (2007). Drugs.

[4] Sajonz P, Natishan T, Wu Y, McGachy N, DeTora D (2005). J Liq Chrom Relat Technolog.

[5] Muratani T, Doi K, Kobayaashi T, Nakamura T, Matsumoto T (2009). Jpn J Antibiot.

[6] Yamada M, Watanabe T, Baba N, Takeuchi Y, Ohsawa F, Gomi S (2008). Antimicrob Agents Chemother.

[7] Kobayshi R, Mami K, Keiko H, Miyuki M, Keisuke S, Kimiko U (2005). Antimicrob Agents Chemother.

[8] Sato N, Kijima K, Koresawa T, Mitomi N, Morita J, Suzuki H, Hayashi H, Shibasaki S, Kurosawa T, Totsuka K (2008). Drug Metab Pharmacokinet.

[9] ICH (2000) Stability testing of new drug substances and products (Q1AR). International conference on harmonization, IFPMA, Geneva

[10] Bakhi M, Singh B, Singh A, Singh S (2002). J Pharm Biomed Anal.

[11] Gaussian 03 (2003) Revision B.05. Gaussian Inc., Pittsburgh.

[12] Cielecka-Piontek J, Krause A, Zalewski P, Lunzer A, Jelińska A (2012). Acta Chrom.

[13] Mantovani L, Sauago C, Camargo V, Dilveva V, Garcia C, Schapoval E, Mednez A (2012). Acta Chrom.

[14] Cielecka-Piontek J, Zając M, Jelińska A (2008). J Pharm Biomed Anal.

[15] Cielecka-Piontek J, Jelińska A, Dołhań A, Zalewski P (2012). Inter J Chem Kin.

